# Efficiency Comparison of Reduced Graphene Oxide (rGO) Composite-Incorporated Polyacrylonitrile Nanofibrous Filters for Respirable Crystalline Silica Dust and Coal Dust

**DOI:** 10.1021/acsomega.5c11145

**Published:** 2026-03-16

**Authors:** Mahsa Moradi, Ashish Kakoria, Lina Zheng, Guang Xu

**Affiliations:** † Department of Mining and Explosives Engineering, 14717 Missouri University of Science and Technology, Rolla, Missouri 65401, United States; ‡ Jiangsu Engineering Research Center for Dust Control and Occupational Protection, 12392 China University of Mining and Technology, Xuzhou 221008, People’s Republic China; § School of Safety Engineering, China University of Mining and Technology, Xuzhou 221008, People’s Republic of China; ∥ Institute of Occupational Health, China University of Mining and Technology, Xuzhou 221008, People’s Republic of China

## Abstract

The health of miners working in underground mines is greatly threatened by their exposure to respirable crystalline silica (RCS) found in coal dust. While coal dust includes RCS, it is important to know the specific filtration efficiency for RCS particles compared to coal dust to improve protective measures. This study compares the filtration efficiency of reduced graphene oxide (rGO) composite-incorporated Polyacrylonitrile (PAN) nanofibrous filters at four different concentrations of rGO (0, 2, 6, and 10 wt%) for RCS particles and coal dust. The filters were made using the electrospinning method. They were tested separately with RCS particles and coal dust with particle sizes from 0.3 to 10 μm, within the respirable range, at various concentrations. Filtration effectiveness was evaluated using a particle counter. A dry dust aerosol generator, model 3410U, operating at 1–6 bar, was used to disperse particles. The results show that the rGO-PAN filters are almost equally effective in removing RCS particles and coal dust across all tested flow rates: 30, 50, and 80 L per minute (L min^–1^). Moreover, at a flow rate of 50 L min^–1^, the 6 wt% rGO-PAN filters showed greater efficiency for both types of dust. This indicates their strong potential for targeting both silica and coal dust particles. This study provides useful insights for creating effective filtration systems to lower health risks linked to RCS exposure in underground mining settings.

## Graphical abstract

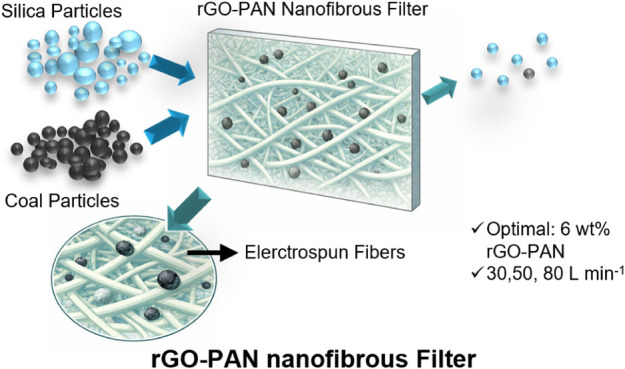

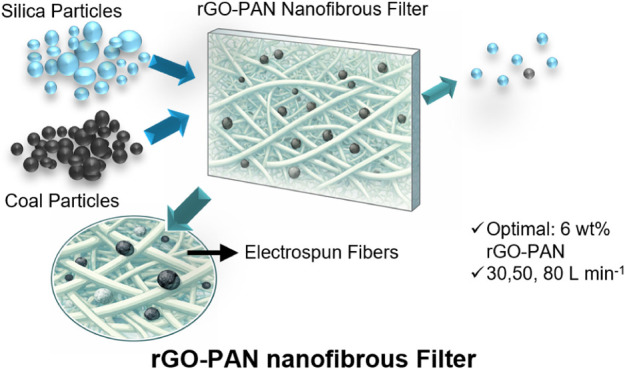

## 1 Introduction

The growing scale of coal mining activities and the generation of significant amounts of coal dust, particularly the dangerous respirable crystalline silica (RCS) it contains, seem to warrant more attention due to the serious health risks they pose to miners. These effects are associated with diseases such as pneumoconiosis, silicosis, fibrosis, and certain cancers.^1–5^ Particular emphasis is placed on respirable dust particles measuring between 0.3 and 10 μm, especially those 5 μm or smaller, as they can infiltrate the respiratory system and penetrate deep into the alveoli and lungs.^6–8^ To reduce exposure, a variety of dust control technologies, such as cyclone dust collectors, electrostatic precipitators, and fibrous surface filters, have been designed and adapted for the specific demands of coal mines.^9–11^ However, even with these technologies, it remains challenging to consistently lower respirable dust levels in miners’ exposure zones to within permissible exposure limits. Therefore, personal protective measures, especially the use of face masks, have become critical in high-risk areas. Improving filtration efficiency is therefore essential for developing advanced filters that can meet the growing need for protection.

In recent years, various polymers have been widely used to produce high-efficiency nanofiber filters, including polyacrylonitrile (PAN),^12,13^ polyvinylidene fluoride (PVDF),^14–16^ poly­(vinyl alcohol) (PVA),^17–19^ polyimide (PI)^20–23^ and polylactic acid (PLA).^24–27^ Notably, extensive research in advanced filtration has primarily focused on polyacrylonitrile (PAN).^28,29^ PAN, with the chemical formula (C_3_H_3_N) and high carbon content, exhibits excellent polymer strength, low density, elasticity, solvent resistance, and the ability to retain its shape during pyrolysis.^29^ Additionally, its surface can be modified or enhanced with additives. Electrospinning technology, widely used for fabricating fiber membranes, is frequently employed with PAN to create robust structures that enhance particle capture.^30^ The physicochemical properties of PAN have been further improved by incorporating various nanomaterials, such as graphene oxide (GO) and reduced graphene oxide (rGO). These materials possess surface functional groups, exceptional mechanical properties, high surface area, and excellent electrical conductivity, making them essential for enhancing a filter’s ability to capture and retain respirable particles effectively.^31^ However, the gap in understanding the use of rGO and PAN is determining the optimal concentration of rGO in PAN for creating a filter that can remove respirable particles in coal mines containing respirable crystalline silica compounds with high efficiency and low-pressure resistance.

We hypothesize that by incorporating rGO into PAN and using the electrospinning technique, we can fabricate new filters with high efficiency for removing RCS and coal dust across various particle size distributions. This could lead to improvements in air quality and safety for miners. We expect the removal efficiency to differ among various dust types because of their unique chemical characteristics. Adjusting the rGO concentration within the PAN matrix to identify an optimal ratio is expected to enhance removal efficiency, enabling the selection of the most effective formulation for practical use. That optimal ratio will also cause a low-pressure drop in the filters. In this hypothesis, the independent variables are dust type (silica and coal), rGO concentration in the PAN membranes, and airflow rate. In contrast, the dependent variables are particle removal efficiency and membrane pressure drop. Previous studies on rGO-PAN nanofibers targeting PM_2.5_ removal have shown that rGO is a more effective additive than GO, as it retains oxygen-functional groups while enhancing the mechanical properties of the membrane. These functional groups, such as hydroxyl (−OH), carboxyl (−COOH), and carbonyl (—C=O), facilitate strong intermolecular interactions, which significantly improve particle capture. Embedding rGO within the PAN matrix not only reinforces the structural stability of the filter but also increases its filtration performance. X-ray photoelectron spectroscopy (XPS) analyses confirm the presence of polar groups (C–O, C=O, C–N) on PM_2.5_ surfaces, which interact synergistically with the polar functionalities in rGO and PAN, boosting the filter’s purification ability.^32^ This highlights the importance of understanding the interaction between filter materials and dust characteristics in achieving high removal efficiency. Additionally, research on PET/SiO_2_/FPU nanofibrous membranes has demonstrated the effectiveness of electrospinning in creating precise, high-performance filters.^27^ Building on these findings, this study employs electrospinning to fabricate rGO-PAN filters and, unlike prior research, uses RCS and coal mine dust dispersed in solid form through an aerosol generator. Filtration tests were conducted across typical respirable airflow rates, offering a more realistic assessment of filter performance under mining conditions.

The purpose of this study is to develop and evaluate rGO-PAN nanofiber filters for the removal of RCS and coal dust, and to identify the optimal rGO concentration in PAN that achieves high filtration efficiency with minimal pressure drop, making it suitable for personal protection in coal mines. Four types of filters were fabricated by blending PAN with varying concentrations of rGO (0, 2, 6, and 10 wt%) through electrospinning. Each was tested under three airflow rates (30, 50, and 80 L min^–1^), reflecting typical breathing patterns.^33^ Filtration performance was measured using an optical particle sizer spectrometer. In this study, among the investigated filters with rGO incorporated into the PAN matrix, the filter containing 6 wt% rGO showed the most favorable balance between filtration efficiency and pressure drop. This behavior was observed for both RCS and coal dust under all examined airflow conditions. These results provide insight into how rGO content, airflow rate, and dust type influence overall filter performance.

## 2 Procedure

### 2.1 Materials

The materials were including PAN powder with the chemical formula C_3_H_3_N, density of 1.184 g.mL^–1^ at 25 °C, and weight-average molecular weight (Mw) of 150,000 g.mol^–1^; rGO powder manufactured in Spain; and N,N-Dimethylformamide (DMF, 99.8% purity) with the molecular formula C_3_H_7_NO, density of 0.944 g.mL^–1^ at room temperature, and a molar mass of 73.09 g.mol^-1^ obtained from Sigma-Aldrich, USA. The Keystone Mineral Black 325 coal powder was chosen as the primary coal-based material. Two types of silicon dioxide powders were used for the silica-based components, ranging from 0.3 to 10 μm. The first type was a silicon dioxide powder with a purity of approximately 99% and a particle size ranging from 0.5 to 10 μm (commonly referred to as white quartz, quartz, and silicon dioxide). The second type was silica fume powder, which has an average particle size ranging from 0.2 to 0.3 μm (also known as Silica, Silicic Anhydride, or Amorphous Silicon Dioxide). Both types of silica powders were sourced from Sigma-Aldrich, USA.

### 2.2 Preparation of rGO-PAN Solutions

For the rGO-PAN filter, 1 g PAN powder was mixed with varying amounts of rGO powder: 0.02, 0.06, and 0.1 g (or 2%, 6%, and 10% weight percentage, respectively). These mixtures were dissolved in DMF to a total volume of 10 mL. The solutions were stirred magnetically (100 rpm) while being heated for 8 h (at 70 °C) to achieve a homogeneous rGO-PAN dispersion. Additionally, only 1 g of PAN powder was used in 9 mL of DMF for the PAN filter.

### 2.3 Electrospinning System

Our electrospinning setup comprises a syringe pump fitted with a 10 mL syringe, a high-voltage power supply, a rotating drum collector, and an acrylic enclosure designed to shield the process from fluctuations in temperature and humidity (Figure 1). A fan placed inside this enclosure promotes rapid and uniform solvent evaporation, stabilizes temperature and humidity, and disperses volatile vapors to ensure both safety and consistent fiber quality. Key parameters affecting nanofiber filter formation include the applied voltage, drum rotation speed, distance between the needle tip and grounded collector, polymer solution concentration, and feed rate.^34,35^

**Figure 1:**
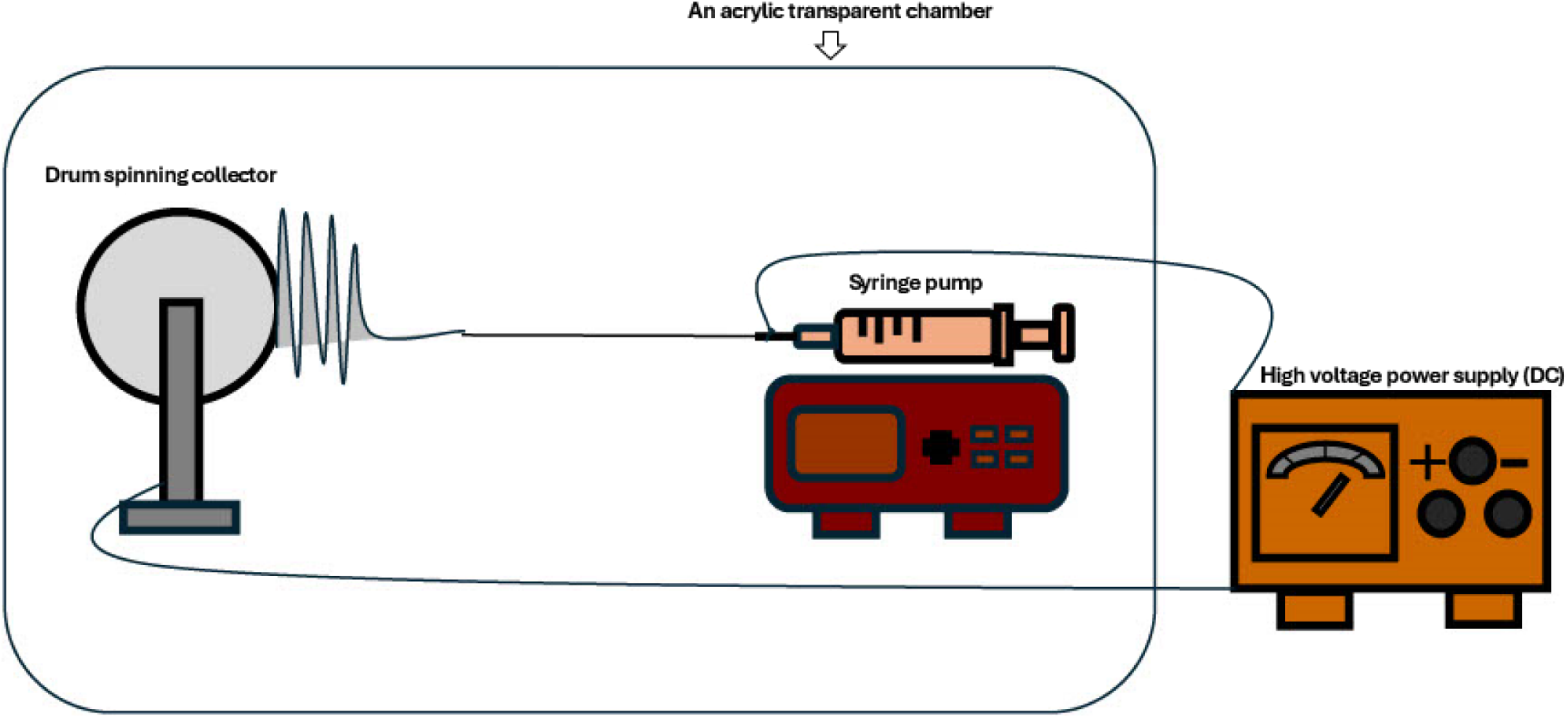
Electrospinning system designed for this study.

In this study, the distance between the needle tip (0.91 mm in diameter and 50.8 mm in length) and the grounded stainless-steel roller (covered with nonwoven polypropylene substrate) was fixed at 15 cm. As the viscosity of the solution increases with higher rGO content, it is essential to adjust the flow rate, roller rotation speed, and voltage. This helps maintain a stable, continuous jet and minimizes droplet formation during electrospinning. To reach that, the roller rotation speeds varied from 185 rpm for solutions containing 0 to 2 wt% rGO to 263 rpm for solutions with 6 to 10 wt% rGO. Also, the solution feed rates for 0, 2, 6, and 10 wt% rGO-PAN were 1, 0.8, 0.4, and 0.4 mL/h, respectively. Additionally, the applied voltage ranged from 13 kV for 0 to 2 wt% rGO-PAN to 18 kV for 6 and 10 wt% rGO-PAN.

### 2.4 Characterization

The morphological features and microstructure of the filter media were analyzed using a Field Emission 600 Helios NanoLab, which integrates a high-resolution field-emission scanning electron microscope (SEM) with a focused ion beam (FIB). This dual-beam system facilitated precise fiber diameter measurements and detailed visualization of fiber arrangement. The SEM’s electron beam captured high-resolution images of the fibers, while the FIB was employed to mill selected regions, revealing cross-sectional features. By correlating these nanoscale observations with fiber uniformity, spatial distribution, and diameter variability, this study provided critical insights into the relationship between structural characteristics and filtration efficiency. Specifically, the findings highlighted how fiber morphology influences particulate capture and flow resistance, underscoring the importance of precise morphological characterization in optimizing filter performance for various applications. The diameter distribution of the nanofibers was determined by analyzing approximately 100 individual fiber diameters from SEM images taken at a magnification of 100,000x, using an accelerating voltage of 18 kV, a milling resolution of 5 μm, and a beam current of 0.17 nA.

### 2.5 Measurement of Filtration Performance

Figure 2 illustrates the experimental setup in this study, which comprised 1) two air compressors, 2) two diffusion dryers, 3) two HEPA filters, 4) a dust aerosol generator (TSI 3410U), 5) a rotameter, 6) a PVC pipe with an inner diameter of 4 in. and a length of 71.8 in., 7) an optical particle sizer spectrometer (condensation particle counter) (TSI 3330), 8) a filter holder, and 9) a filter. The apparatus featured the pipe segmented into two parts, with a filter holder designed to accommodate a 10 cm (3.94 in.) filter having a net face area of 78.54  cm^2^ (12.2 in^2^). Compressed air, initially purified using HEPA filters (TSI Capsule Filter, with a 99.97% efficiency for particles larger than 0.3 μm), was introduced into the test chamber to dilute the dust particles and supply the dust aerosol generator. The dust aerosol generator was employed to disperse two types of test aerosols including (i) silica dust and (ii) coal powder at a dosing speed of 50%, operating at 1–6 bar, and a mass flow rate of 0.7 g/h ranging in size from 0.3 to 10 μm. Additionally, the flow rate of the compressed air was adjusted by the calibrated rotameter to three levels: 30, 50, and 80 L min^–1^.

**Figure 2:**
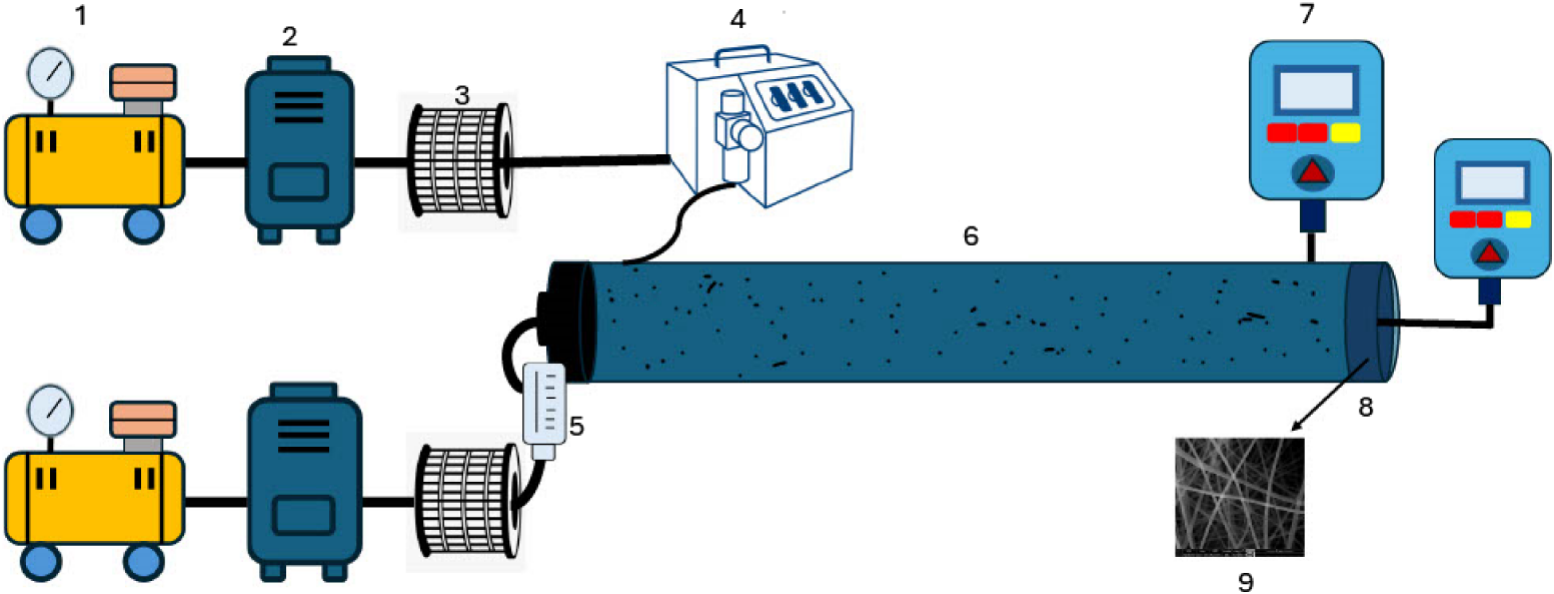
The schematic of the experimental apparatus. 1) Air compressor. 2) Air dryer. 3) HEPA filter. 4) Dust aerosol generator (TSI 3410U). 5) Rotameter. 6) Pipe. 7) Optical particle sizer spectrometer (TSI 3330). 8) Filter holder. 9) Filter.

Nanofiber filters with a 10 cm diameter were sandwiched between two layers of cotton cloth (each with a basis weight of 85 g per square meter) to enhance ease of handling during the assessment of filtration performance. The entire assembly was evaluated to determine the particle filtration efficiency (PFE, denoted as η). Particle concentrations were measured separately at the upstream and downstream locations, each for a duration of 1 min, using an optical particle sizer spectrometer operating at a fixed sample flow rate of 1 L min^–1^. The 1 min measurement was considered based on the NIOSH 42 CFR Part 84 to evaluate the effect of the filter’s morphology on initial particle removal efficiency and pressure drop under clean media conditions. This approach excludes the influence of cake formation and clogging and was used to determine the optimal rGO concentration in PAN nanofibrous filters.

The filtration efficiency was calculated based on the difference between the upstream and downstream particle concentrations, corrected by subtracting the background particle concentration measured with the cotton cloth, as shown in eq 1

1 $$\mathrm{PEF}\left(\%\right)=\left(1-C_{\mathrm{down}}/C_{\mathrm{up}}\right)\times 100$$

where PEF represents particle filtration efficiency, *C*_up_ (L min^–1^) and *C*_down_ (L min^–1^) are, respectively, upstream and downstream.

This systematic comparison provides insights into the optimal rGO-PAN for enhanced filtration, as well as the influence of airflow rate and dust type on overall filter performance. To better understand the performance of the filters, their quality factor (QF) was calculated using eq 2;

2 QF=−ln(1−η)/(△P)

where η represents filtration efficiency, indicating the fraction of particles removed, and Δρ denotes the pressure drop across the filter, reflecting airflow resistance. A higher QF signifies a filter that efficiently captures contaminants while maintaining low energy loss, making it a crucial parameter for evaluating filtration performance.^36^

The porosity of the nanofibrous filters was determined using the following equation:

3 $$\mathrm{Porosity}=\left(D_{0}-D_{1}/D_{0}\right)\times 100$$

*D*_0_ represents the density of raw PAN and rGO-PAN, while *D*_1_ specifies the density of fibrous filters.

## 3 Results and Discussion

Figure 4e–h shows the SEM images of the fiber filters analyzed to investigate the fiber diameter distribution for each sample. The analysis reveals that the average fiber diameter decreased with increasing concentrations of rGO incorporated into PAN, as illustrated in the chart in Figure 4 (i-l). The average fiber diameters measured for PAN, 2, 6, and 10 wt% rGO-PAN are 527, 507, 469, and 301 nm, respectively. Compared to plain PAN fibers, the average fiber diameters decrease 3.79%, 11%, and 42.9% for 2, 6, and 10 wt% rGO-PAN fibers, respectively. The 10 wt% rGO-PAN filters exhibit the minimum fiber diameter of 287 nm, while the PAN filters show the maximum fiber diameter of 596 nm, corresponding to the thinnest and thickest fibers among the filters. As the fibers became thinner, the overall filter density increased, leading to a decrease in the pore size between fibers.^34^ This reduction in pore size contributes to an improvement in particle capture efficiency, as observed in the experimental results. Moreover, the formation of thinner fibers enhanced the available surface area for particle interception, thereby improving the filtration performance by providing more sites for airborne particle attachment.^37,38^

The results agree with previous observations and indicate that increasing the rGO concentration in PAN consistently enhances filtration efficiency for both coal dust and silica dust, with a slightly greater improvement observed for coal dust, as shown in Figure 4 (a-d). One reason may be that the rGO in the filters contains functional groups such as −COOH and −OH, which enhance its ability to attract and hold particles.^31^ The surface of RCS contains silanol (Si–OH) groups, and coal dust particles possess various oxygen-containing functional groups.^39,40^ Both types of groups can interact with rGO functional groups, which facilitates particle retention. Additionally, Figure 5 shows the distribution of RCS and coal dust particle concentration upstream and downstream of the filters at different flow rates (30, 50, 80 L min^-1^). It shows a significant reduction in the concentration of particles with more than 2 μm in size (near the complete reduction) for all filters at different flow rates. Furthermore, most particle concentrations upstream are related to less than 1 μm. Efficiency rises as rGO loading increases and reaches its peak at an airflow of 50 L min^–1^; beyond this point (at 80 L min^–1^) the efficiency begins to decline, a trend that accords with previous findings.^41^ The 6 wt% rGO-PAN membrane exhibited the highest overall performance, delivering 100% removal of particles ≥ 8.032 μm at 50 L min^–1^. Its lowest measured efficiency, 92% for silica dust in the 0.3–0.374 μm range at 30 L min^–1^, still exceeded the corresponding efficiencies of the other membranes under identical conditions. Moreover, as the rGO content increases, the difference between silica and coal dust removal at a given flow rate narrows; for example, the 2 wt% rGO-PAN membrane achieves efficiencies of 82% (silica) and 83% (coal dust) for 0.300–0.374 μm particles at 80 L min^–1^.

Porosity serves as a critical structural parameter in nanofibrous filters, directly affecting both particle capture efficiency and airflow resistance. Table 1 presents the primary structural characteristics of the nanofibrous filters, including mean fiber diameter, porosity, and membrane thickness. As rGO content increases, both membrane thickness and overall porosity increase, while the average fiber diameter decreases. These trends, indicating smaller pore sizes, would typically suggest that the 10 wt% rGO-PAN filter should exhibit higher filtration efficiency and possibly a slightly higher pressure drop than the 6 wt% rGO-PAN filter. Contrary to this expectation, the filtration results in Figure 4 demonstrate that although the 10 wt% rGO-PAN filter exhibits a higher pressure drop (Table 2), its filtration efficiency is lower than that of the 6 wt% rGO-PAN filter. SEM images in Figure 3 elucidate this discrepancy by revealing pronounced bead formation and increased morphological nonuniformity in the 10 wt% rGO-PAN filter. This is attributed to the combined effects of increased solution conductivity resulting from higher rGO loading and the elevated applied voltage necessary to maintain electrospinning stability, both of which promote jet instability and bead formation. The resulting bead-induced defects introduce localized structural heterogeneities that disrupt the otherwise dense fibrous network, thereby creating bypass flow pathways that diminish effective particle capture.

**Table 1:** Characteristics of the Membranes

Nanofibrous Filters	Fiber Diameter (nm)	Porosity (%)	Thickness (μm)
PAN	527±44.8	77.5	100
PAN-2 wt% rGO	507±30.52	83.7	120
PAN-6 wt% rGO	469±55.2	92.4	155
PAN-10 wt% rGO	301±11.46	93.6	156

**Table 2:** Pressure Drop of PAN and rGO-PAN Nanofibrous Filters at Different Flow Rates

	Pressure drops (Pa)							
	PAN		2 wt% rGO-PAN		6 wt% rGO-PAN		10 wt% rGO-PAN	
Flow rate (L min^–1^)	Silica Dust	Coal Dust	Silica Dust	Coal Dust	Silica Dust	Coal Dust	Silica Dust	Silica Dust
30	10	0	12	5	35	10	35	10
50	40	20	50	31	50	32	125	34
80	40	22	60	31	148	35	250	40

**Figure 3:**
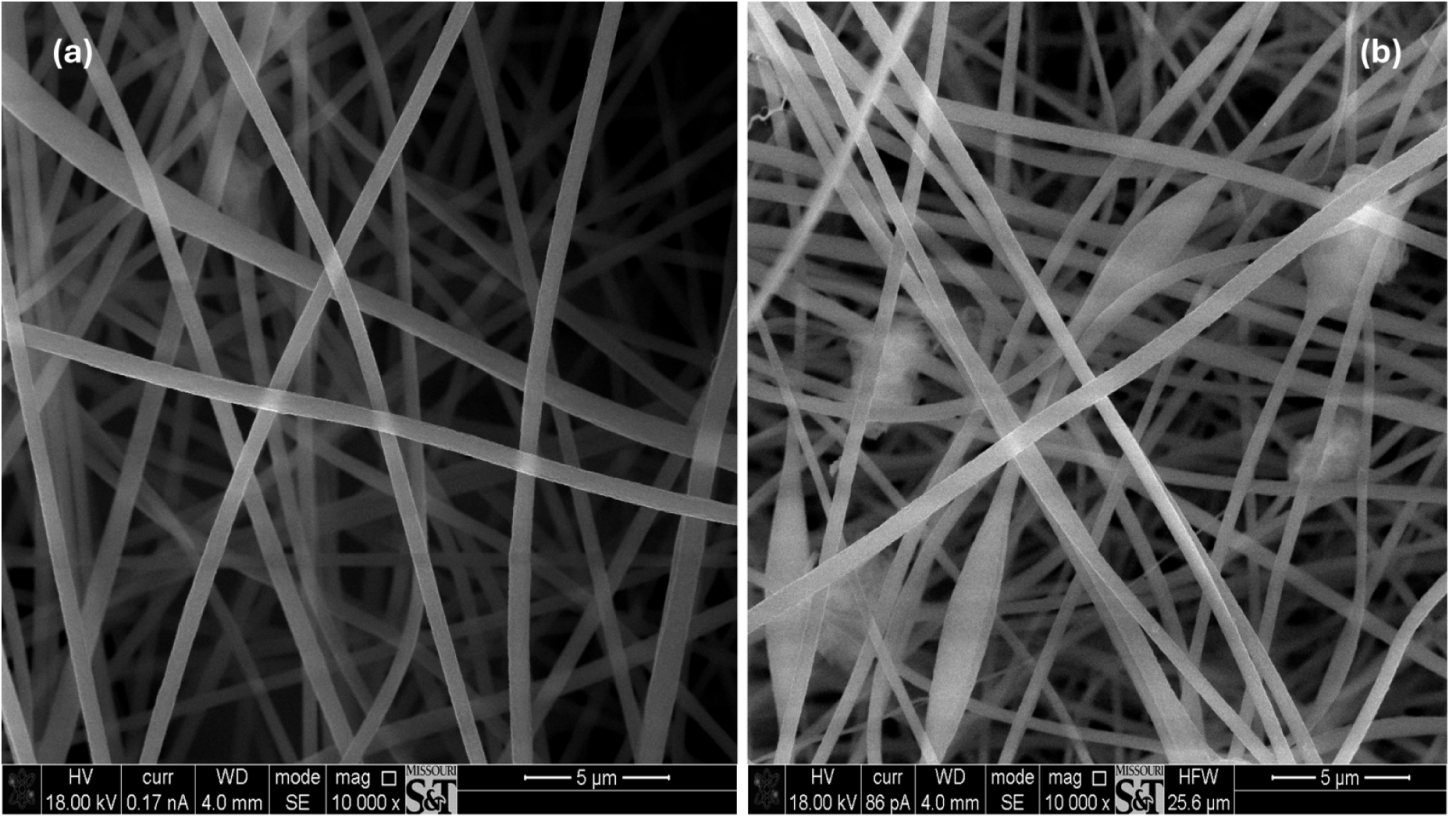
SEM images of electrospun nanofibrous filters: (a) 6 wt% rGO-PAN filter and (b) 10 wt% rGO-PAN filter.

Table 2 presents the pressure drop across filters tested with silica and coal dust, demonstrating the influence of varying particle characteristics on pressure resistance within filters. NIOSH 42 CFR Part 84 specifies that, for filter testing at a face velocity of 5.3 cm s^–1^, the acceptable pressure drop must not exceed 343 Pa. In this study, the filters were evaluated at face velocity of 6.37–17 cm s^–1^. As shown in Table 2, all filters exhibited pressure drop below 343 Pa for both particle types. The highest pressure drop (250 Pa) was observed for the 10 wt% rGO-PAN filter at the highest flow condition during silica dust testing, which is likely attributable to its reduced porosity and smaller pore size, as well as differences in particle characteristics between silica and coal dust. Also, under the same operating conditions, the pressure drop during coal dust testing was substantially lower. These results indicate that the PAN and rGO-PAN nanofibrous filters provide acceptable breathing resistance for both silica and coal dust particles when benchmarked against the NIOSH criteria. However, across all filters and face velocity rates, the pressure drop was higher for silica dust than coal dust.

Quality factor analysis offers insight into the relationship between filtration efficiency and pressure drop. At a 30 L min^–1^ flow rate, we observe larger QF values, which are associated with lower pressure drops; however, as mentioned before, this flow rate results in lower efficiency, as shown in Figure 4 (m-p). Additionally, with increasing flow rates (50 and 80 L min^–1^), which lead to higher efficiency, 6 wt% rGO-PAN exhibits a greater QF for both types of particles (silica and coal dust) compared to 2 and 10 wt% rGO-PAN filters. Therefore, it is evident that 6 wt% rGO-PAN filters exhibit high efficiency at this flow rate, accompanied by a good QF, which is notable due to the acceptable pressure drops compared to other filters. This performance is attributed to a well-balanced fiber structure, which provides an optimal pore size that enables efficient particle capture without inducing excessive pressure drop. The results also highlight the impact of different particulate materials on QF. Across all flow rates, silica dust consistently resulted in lower QF values compared to coal dust, although the difference was marginal yet noticeable. Additionally, efficiency curves demonstrate that coal dust was removed more effectively across all filters and flow rates (Figure 5). This suggests that material properties play a role in filtration efficiency, affecting both particle capture effectiveness and overall filtration performance.

**Figure 4:**
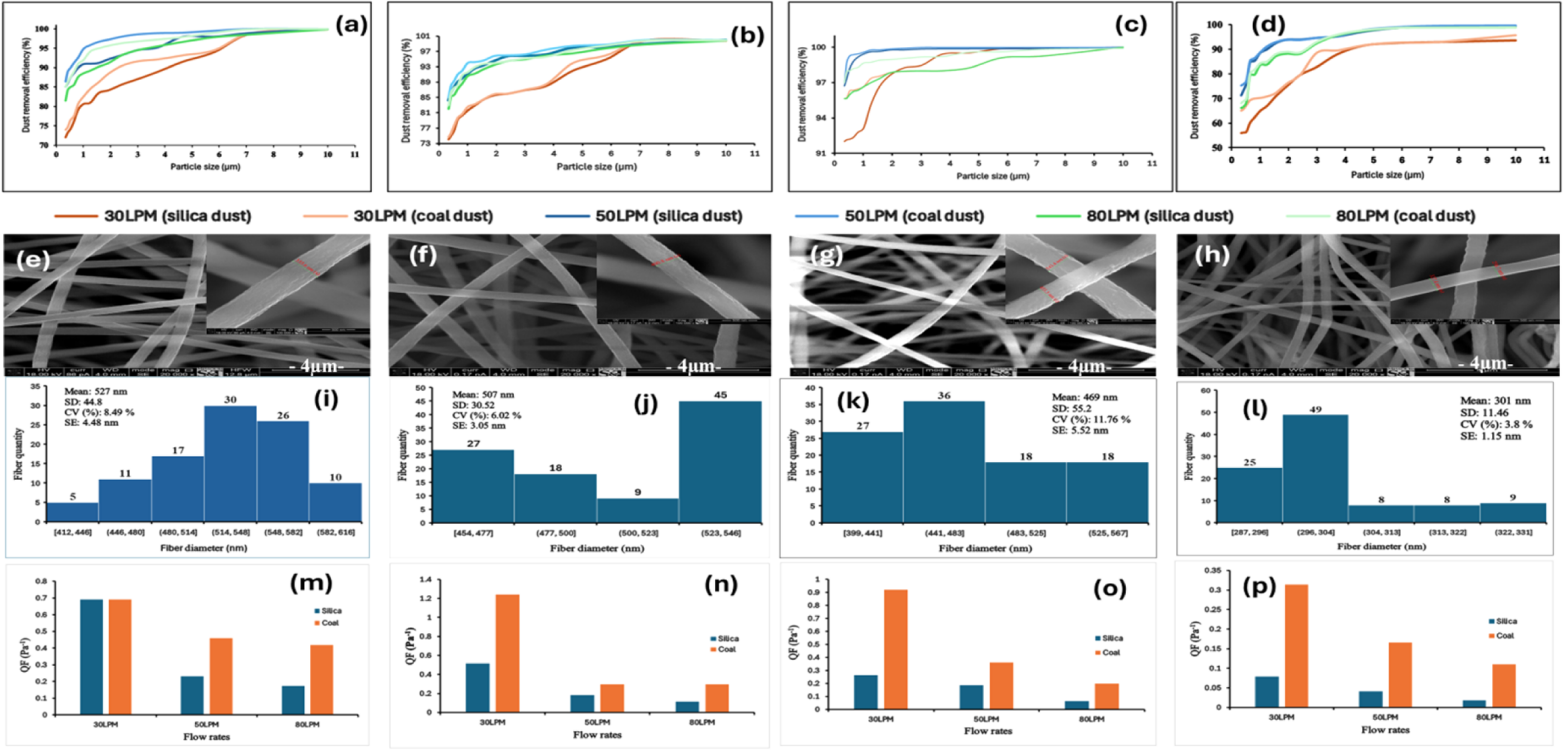
Comprehensive of PAN and rGO-PAN membranes for silica and dust filtrations. (a–d) Dust removal efficiency (%) as a function of particle size for neat PAN (a), 2 wt% rGO-PAN (b), 6 wt% rGO-PAN (c), and 10 wt% rGO-PAN (d) membranes, tested at three airflow rates (30, 50, and 80 L min^–1^). (e–h) SEM images of the corresponding membranes: (e) PAN, (f) 2 wt% rGO-PAN, (g) 6 wt% rGO-PAN, and (h) 10 wt% rGO-PAN. Main panels at 20,000× magnification; insets show fiber morphology at 100,000× magnification (scale bars: main panels, 4 μm; insets, 500 nm). (i–l) Fiber diameter distributions obtained from image analysis of the SEM images in panels (e–h): (i) PAN, (j) 2 wt% rGO-PAN, (k) 6 wt% rGO-PAN, and (l) 10 wt% rGO-PAN. (m–p) Quality factor QF = −ln­(1 – η)/ΔP for silica (blue) and coal dust (orange) at airflow rates of 30, 50, and 80 L min^–1^, calculated for (m) PAN, (n) 2 wt% rGO-PAN, (o) 6 wt% rGO-PAN, and (p) 10 wt% rGO-PAN membranes. Higher QF values indicate a more favorable balance between filtration efficiency and pressure drop.

**Figure 5:**
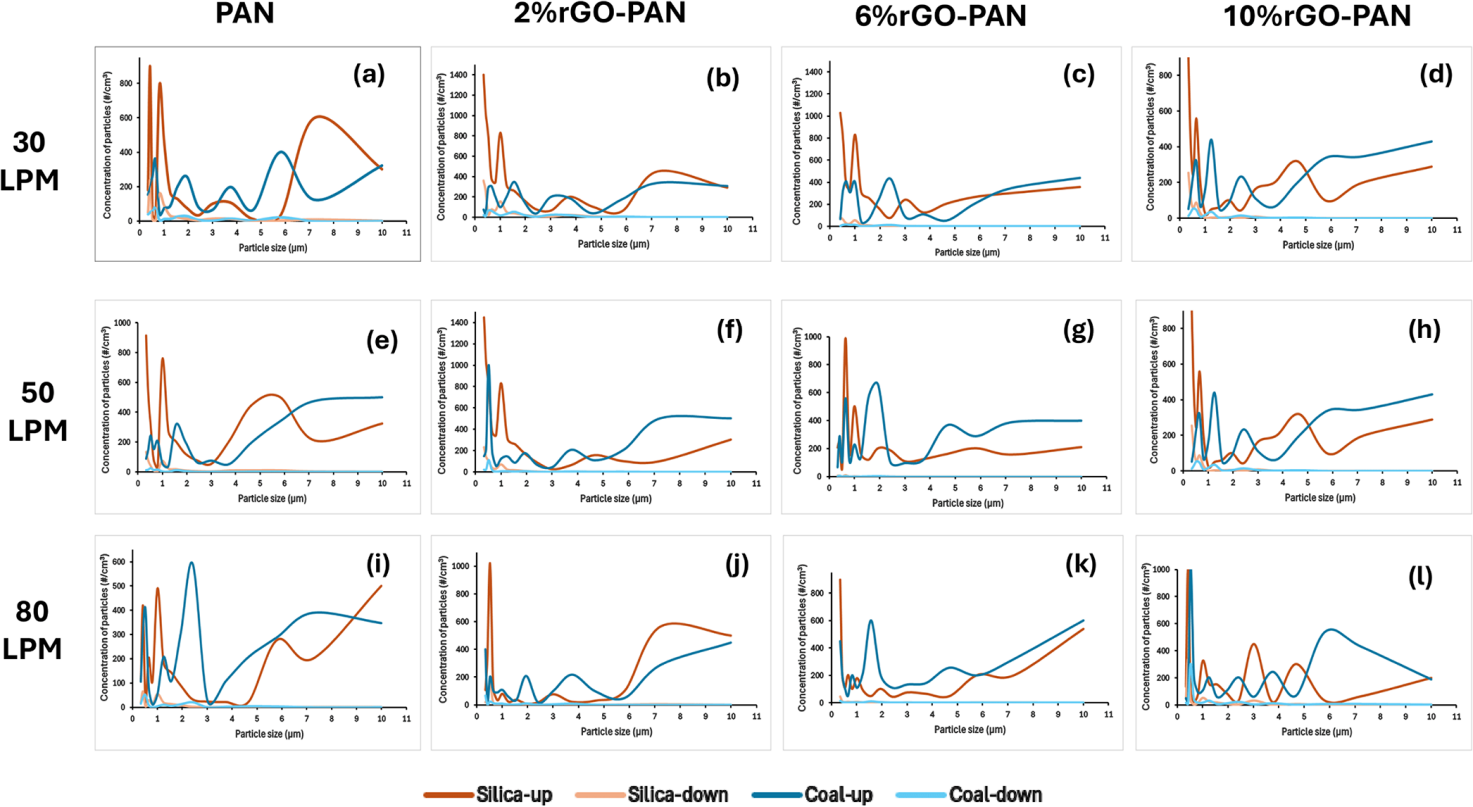
Particle number concentrations upstream and downstream of the filters in the different flow rates.

## 4 Conclusion

In this study, reduced graphene oxide (rGO) was successfully incorporated with polyacrylonitrile (PAN) to create new nanofibrous filters for the removal of coal and RCS particles in mines. The filters containing 0, 2, 6, and 10 wt% rGO were fabricated and tested for particle sizes ranging from 0.3 to 10 μm, including both RCS and coal dust, at three flow rates (30, 50, and 50 L min^–1^) by using the electrospinning method. Taking SEM images allowed us to understand the structure of the filters and their effects on their performance.

The results showed that increasing the concentration of rGO in PAN causes a decrease in diameter and pore size in the filters. The 6 wt% rGO in PAN provided the best outcome, as it has a good balance between efficiency and low-pressure resistance. At 50 air flow, a 6 wt% rGO-PAN filter achieved nearly 100% removal for particles above 1 μm and approximately 97% for particles smaller than 1 μm. A study that used PAN for making filters showed that the efficiency ranged from 94.83% to 99.6% for aerosol particles with diameters around 0.3 μm.^42^ This clearly indicates that adding rGO to PAN in our research increased the efficiency of the filter for this range of particles, especially at 6 wt% rGO. Across all flow rates and filters, coal dust was removed more effectively than RCS. It demonstrates the impact of chemical dust particles on the filter’s capture efficiency. Additionally, all filters demonstrated quality factors above 0.03 Pa^–1^, which other studies considered a reasonable value, confirming their strong potential for working effectively at low-pressure resistance.

These results demonstrate the novelty of these filters and their practicality for enhancing safety in mines. The filters have the merit of not only providing good efficiency removal for RCS and coal particles but also maintaining acceptable pressure resistance. Therefore, they will be an excellent choice for use in both dry scrubbers and personal protective masks. The study fills a gap related to using the great features of these polymers and chemicals for our goal of removing the RCS and coal dust, which offers clear insights that extend beyond general particulate matter studies.

Nevertheless, certain limitations should be acknowledged. The tests were conducted under controlled laboratory conditions, with air flows comparable to those used in the mask tests and a fixed duration of 1 min. Future studies should investigate a broader range of airflow rates and exposure durations. Additionally, further characterization is necessary to clarify the chemical interactions between (silica and coal dust) particles and rGO-PAN filter surfaces.
